# Bioinformatics: Literature Searchlight

**Published:** 2004-11

**Authors:** W. Conard Holton

The time and cost required to bring a new drug to market can exceed 10 years and $800 million, according to the Tufts Center for the Study of Drug Development. Now researchers at etexx Biopharmaceuticals in Dallas, Texas, are using software that they say will slash this time and cost. How? By mining the medical literature for hints on new uses for drugs already approved by the Food and Drug Administration.

Repurposing existing drugs can offer substantial payoffs for both pharmaceutical companies and the public. For example, bupropion has been licensed separately as an antismoking drug (Zyban) and an antidepressant (Wellbutrin); Rogaine, now used to treat hair loss, was originally developed as a treatment for high blood pressure.

The software was created at The University of Texas (UT) Southwestern Medical Center by biochemistry professor Harold Garner and colleagues, and has been licensed to etexx, which Garner founded. Known as IRIDESCENT (for **I**mplicit **R**elationship **IDE**ntification by in-**S**ilico **C**onstruction of an **E**ntity-Based **N**etwork from **T**ext), the program allows full-scale automated analysis of records in MEDLINE, the National Library of Medicine’s bibliographic database. Eventually the software could be used with other online resources such as the *Physicians’ Desk Reference* and even internal documents from pharmaceutical and biotech companies.

The software analyzes MEDLINE abstracts to identify and evaluate statistical relationships among biomedical terms such as names of genes, phenotypes, drugs, and diseases. The program compares how often sets of these terms appear in texts relative to random probability. It can identify and compare over 300,000 different biomedical terms along with their spelling variations, synonyms, and acronyms. A network of these “co-mentions” is created and then analyzed by a statistical program to find indirect or implicit connections.

IRIDESCENT then scores the objects for relevance, significance, and interest, allowing the researcher to inspect the resulting connections to trigger hypotheses on new uses for existing drugs. The team showed the value of this approach by validating in several lab trials a connection between the drug Thorazine, used to treat psychotic disorders, and a reduction in the progression of cardiac hypertrophy, or enlargement of the heart, which the program had predicted. The results were published 12 February 2004 in *Bioinformatics*.

Besides conducting its own lab research on potential repurposed drugs, etexx will help pharmaceutical and genomics organizations sort through existing data and generate hypotheses from high-throughput data processes such as microarrays or proteomics mass spectroscopy analysis. “Everyone is trying to find ways to develop drugs cheaply,” says Stephen Johnston, director of the UT Southwestern Center for Biomedical Inventions, which develops new drugs and procedures. “Garner has had very promising preliminary results.”

